# Surface temperature behavior in view of the conversion of tropical dry forest into anthropic uses, northern Minas Gerais–Brazil

**DOI:** 10.1371/journal.pone.0270991

**Published:** 2022-07-27

**Authors:** Lucas Augusto Pereira da Silva, Andre Medeiros Rocha, Claudionor Ribeiro da Silva

**Affiliations:** 1 Institute of Geography, Program of Post-Graduation in Geography, Federal University of Uberlândia, Uberlândia, Minas Gerais, Brazil; 2 Program of Post-Graduation in Physical Geography, University of São Paulo, São Paulo, São Paulo, Brazil; Oregon State University, UNITED STATES

## Abstract

Tropical dry forests (TDFs) are essential for environmental dynamics, especially in terms of climate variations. However, several anthropic factors have threatened the integrity of TDFs, and consequently the surface temperature (ST), which is a proxy variable for several environmental processes in TDFs. So, understanding their behavior is crucial. The objective was to analyze the behavior of surface temperature owing to conversion of TDFs into anthropic uses in northern Minas Gerais between 2007 and 2016. In 9 years, dry forests decreased by 22.9%, with pastures as the central driver (counted 93% of change). Between 2007 and 2016, there was an increase in ST by 1.55 K ± 1.15 K. When TDFs were converted to pastureland, the increase in ST was 2.21 K ± 1.39 K and for crops by 0.57 K ± 1.24 K. The remaining TDFs (2016) had an increase in their thermal average of 1.41 K ± 1.02 K. This analysis is essential for the adoption of conservation actions for the maintenance of ecological corridors in TDFs, considering their importance in the ecosystem context.

## Introduction

Subtropical and subtropical and tropical dry broadleaf forests are the most endangered forested biome on the globe [1, 2] and although such fact has ever been known in a form of an alert concerning its conservation status and level of threatening by Janzen [3] in the 1980s, still nowadays, more than 30 years after, tropical dry forests (TDFs) preservation and importance remains a debate topic [4].

As a general incentive to the intense anthropic pressure observed in tropical dry forest areas, its physiological and ecological characteristics are among the main reasons that make it a place favorable to human occupation when compared to the savannas and rain forests domains. TDFs generally occupy flat areas, in fertile soils, with low aluminum content and present physiological and ecological behavior adapted to seasonal droughts [5]. Savannas, on the other hand, occupy more poor, acidic soils, depending on the subtype, and with high aluminum content [6].

Compared to rain forests, dry forests have lower stature, and their ecological processes are time-limited due to its property of deciduousness associated with seasonality. Its net primary productivity, for instance, is lower when compared to rain forests, since TDFs photosynthetic activity is tied to the rainy season [7]. These characteristics of seasonality, fertile soils, and relatively flat areas are among the factors that make tropical dry forests prone to human activity occupation, in particular agriculture, whose agricultural calendar lifestyle in general better fits with [8].

In terms of conservation, subtropical and tropical dry broadleaf forests ecoregion with more than 50% of their area in protected status (half protected status) accounts only for 2 out of the 56 (3.6%) total existing ecoregions, while 26 of them (46.4%) shows less than 20% in protected status (nature imperiled status) [2]. The aforementioned fact has a relative weight and constitutes a matter of concern, with a view that combined effect of low degree of protection and high anthropic pressure associated with dry forests put at risk the maintenance of ecosystem services those environments provide. TDFs, for example, are largely characterized by providing important ecosystem services such as: carbon storage source [9, 10], biodiversity wealth [8] and hydrological [11] and energy-balance [12–14] regulation.

The disappearance of the listed ecosystem services earns notoriety because such processes interfere with the global air [15] surface [16] temperature, which are parametric variables for measuring the average climatic state of the Earth. In this way, the changes in land use and land cover associated with deforestation and greenhouse gas emissions (GGEs) constitute exemplary anthropogenic radiative forcings that impact global temperature, producing increase (positive forcing) or reduction (negative forcing) [17].

The loss of forest areas, on the one hand, has direct effects on carbon cycle. Forests are one of the largest terrestrial carbon sinks [9], acting as one of the strategies for balancing atmospheric CO_2_ content. The replacement of forest by pasturelands or croplands makes such carbon sequestration strategy an unfeasible plan [18]. On the other hand, the loss of forested areas promotes reduction on evapotranspiration rate and surface storage of precipitation. In terms of radiation balance, the deforestation of forest areas has also the effect of increasing the surface albedo and temperature and decreasing surface latent and sensible heat transfer capacity [11–13].

In the context of TDFs, Brazil stands out for sheltering three of the most extensive ecoregions of the neotropics and by such role played, preservation of Brazilian tropical dry forests is of extremely concern. In terms of forest resources, Brazil occupies the second largest forest area on the planet, encompassing 12% of global forests resources (app. 496 million ha) for period 2010–2020 and impressively is the country with the highest annual average loss of forests areas (app. -1.4 million ha /yr) [19]. On the other hand, in terms of dry forests, Brazil stands out in the neotropics for harboring the two ecoregions of TDFs with a larger extent of areas at least degree of protection (nature imperiled category) [2].

In view of the ecosystem services that TDFs play and considering the role that Brazil has in dry forest resources, the current investigation aims to understand the behavior of the surface temperature in the north of Minas Gerais before and after the TDFs conversions to anthropic uses between 2007 and 2016. For this purpose, remote sensing techniques were employed, in order to allow the spatial analysis of extensive areas, to have fast and continuous imaging of the surface, and, mainly, to make products available free of charges. Of equal importance, it is emphasized that remote sensing has often been used as a tool for monitoring and modeling aspects of vegetation [20–22].

## Materials and methods

### Study area

**Fig 1:**
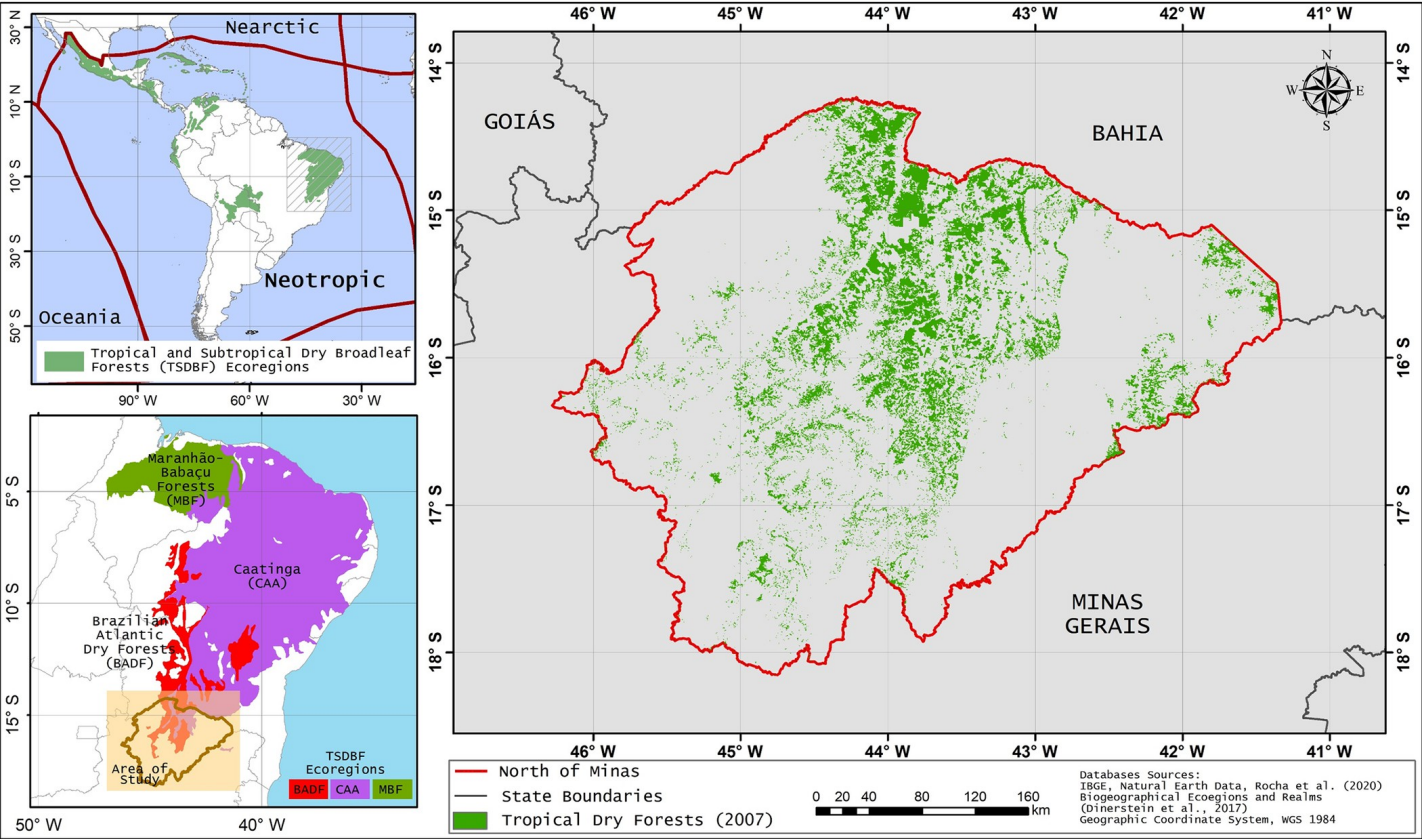
Location of TDFs in the north of Minas Gerais, Brazil, South America.

The study area comprises the southern sector of the ecoregions: Caatinga and Brazilian Atlantic dry forests, two of the 56 ecoregions that compose the Tropical and subtropical broadleaf dry forest biome–neotropic realm (Fig 1). Such area was chosen mainly by 1) possessing historic of TDFs conversion to anthropic uses [23, 24], 2) status of nature imperiled in the categorization scheme of levels of protection for ecoregions presented by Dinerstein et al. [2], 3) compose two of the four most extended ecoregions areas of TDFs in the neotropics, 4) encompassing areas with a high level of annual carbon sequestration [25]. In view of those criteria, the north of the state of Minas Gerais was taken as an area of investigation, being situated between latitudes 14° and 18°S, longitudes 46° and 42W°.

The TDFs had an area of 17.480 km^2^ in 2016, distributed over all extensions of the study area. The climatic context of the study area is marked by two climatic types, Aw (wet tropical, with dry winters) and BSh (semi-arid). The rainfall patterns for the region are marked by dry winters and rainy summers, ranging from 700 to 1200 mm, and average temperature ranging from 294 K to 298 K [26]. Morphoclimatically, the area falls within the transition bands between Cerrados and Caatingas [27]. The relief is flat to slightly undulating, composing extensions of the São Francisco valley. The area is predominantly marked by oxisols, nitosols, and neosols [28].

### Database and processing

For the spatial representation of anthropic and natural domains, we used the products provided by Rocha et al. [24], derived from the mapping of land uses and land cover between the north of the state of Minas Gerais and the south of Piauí (the northeast portion of Brazil) for 2007 and 2016. To map the forests in this portion of Brazil, the authors used the MOD13Q1 products (250 meters spatial resolution) from the MODIS sensor [29]. The modeling was done from supervised classification with the decision tree algorithm [30]. Based on validation processes, the authors obtained an overall accuracy of 89.40% and a kappa coefficient of 86.79% [24].

Originally, the mapping by Rocha et al. [24] has six classes (TDFs, crops, pastures, urban areas, water bodies, and other vegetation), however, for our study, only three were used (TDFs, crops, and pastures). Rocha et al. [24] obtained commission and omission errors for the land use and land cover classes, in which TDFs had 15% and 26.84%, pastureland 28% and 19.86%, and crops 6.4% and 3.14%, respectively.

The MOD11A1 product (tiles H13V10 and H14V10, with a spatial resolution of 1 km) [31] from the MODIS sensor was used to represent the surface temperature dynamics in the study area. This product was provided by the United States Geological Survey. To contemplate our approach, 365 images were obtained (daily time scale) for the two years analyzed (2007 and 2016). The MOD11A1 is a reliable and accurate product for the values of ST because in its generation it considers factors such as i) emissivity, ii) water vapor content in the atmosphere and iii) removal of pixels contaminated by clouds, which is essential for estimation of ST [32].

### Analysis

The territorial limit of the 2007 TDFs was used as a spatial clipping for the subsequent analyzes of this study. Although there may have been a change in the coverage of TDFs before 2007, due to the absence of detailed mappings in the domains of TDFs, we assumed this period to establish the natural domain of TDFs. Therefore, all MOD11A1 images were clipped from the defined limit. These procedures were performed in the QGis software [33].

To understand the dynamics of ST in land uses and land cover on a monthly scale, the images were compiled in 12 products (January to December) for the two years. In order for record the annual changes in ST in the study area, we obtained the annual averages for 2007 and 2016. To carry out the statistical analyses, we obtained sample points (distance of 1 km between the points) considering the classes of land uses and land cover (Fig 1B). In 2007, for the TDFs domain, 1,500 points were sampled. In 2016, for TDFs and pastures, 1,000 points were obtained, and for crop 123. A descriptive statistical analysis was performed with boxplot plots from the constructed matrix. These analyzes were performed in the R software with the ggplot2 package [34, 35].

In order to obtain the thermal differences by land use and land cover classes, as well as to understand the patterns of increases and decreases and variability of the ST in the TDFs domain, we calculated the difference between the annual STs (Equation 01).


ΔST=ST16−ST07

Where ΔST is the difference between the temperatures for the years 2016 (ST16) and 2007 (ST07), respectively.

## Results

### Dynamics of land use and coverage in the domain of dry forests

**Fig 2:**
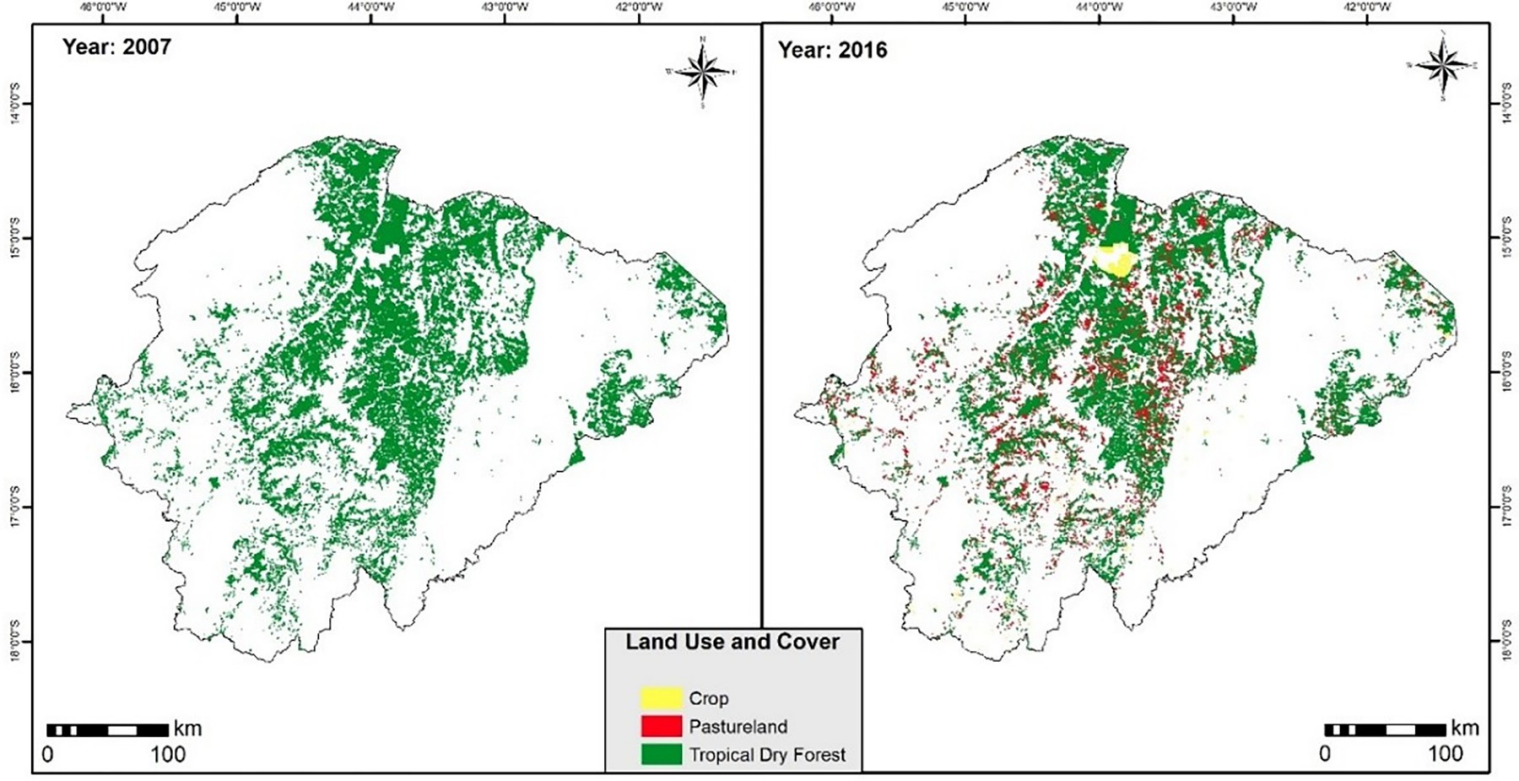
TDFs and anthropic uses, 2007 and 2016, north of Minas Gerais.

The natural domain of TDFs in 2007 had an area of 17,480 km^2^, with distribution in all parallels of the northern region of Minas Gerais (Fig 2). For the period of 2016, there was a loss of forests, leading to a decrease of 22.9% (4,010.6 km^2^) in the natural domain of TDFs, in a short interval of 9 years (Fig 2). The main uses that replaced forests were, mainly, environments with anthropogenic influence. In this case, pastureland systems (3,730.6 km^2^) and agricultural crop (273.6 km^2^). Pastureland counted for 93% of changes in land use and cover in the TDFs domain.

### Spatial distribution of the surface temperature in the northern Minas Gerais context

**Fig 3:**
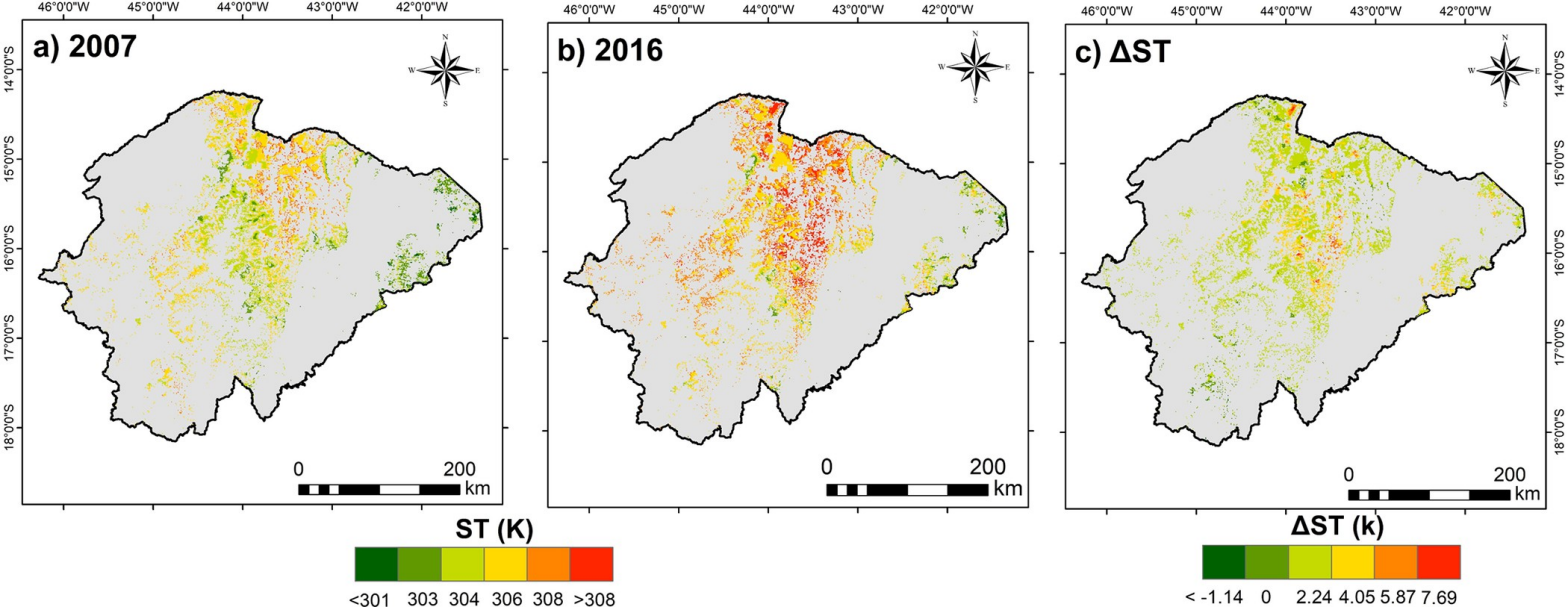
Spatial distribution of surface temperature (ST) in the TDFs domain. a) Spatial distribution of ST for 2007; b) Spatial distribution of ST for 2016; c) Difference (ΔST) between the ST for the years 2016 and 2007 (ΔST = 2016–2007).

For the spatial domain of TDFs in 2007, the ST mean was 305 K ± 1.61 K, ranging between 301 K to 310 K (Fig 3A). In the 9-year interval (2016, Fig 3B), there was an increase of the order of 1.55 K ± 1.15 K in the mean of ST, with maximum values reaching > 7 K (Fig 3C). Considering the changes in the spatial variation of ST between 2007 and 2016 (ΔST), most of the study area (> 70%) showed an increase up to 2.24 K.

In 2016 (Fig 3B), advances were observed in the maximum values of ST (> 308) in relation to the base period (2007). It is possible to infer a relationship between this behavior with the insertion of anthropic uses, mainly pastureland, as this class has advanced to the detriment of TDFs.

### Surface temperature in tropical dry forest and anthropic uses

**Fig 4:**
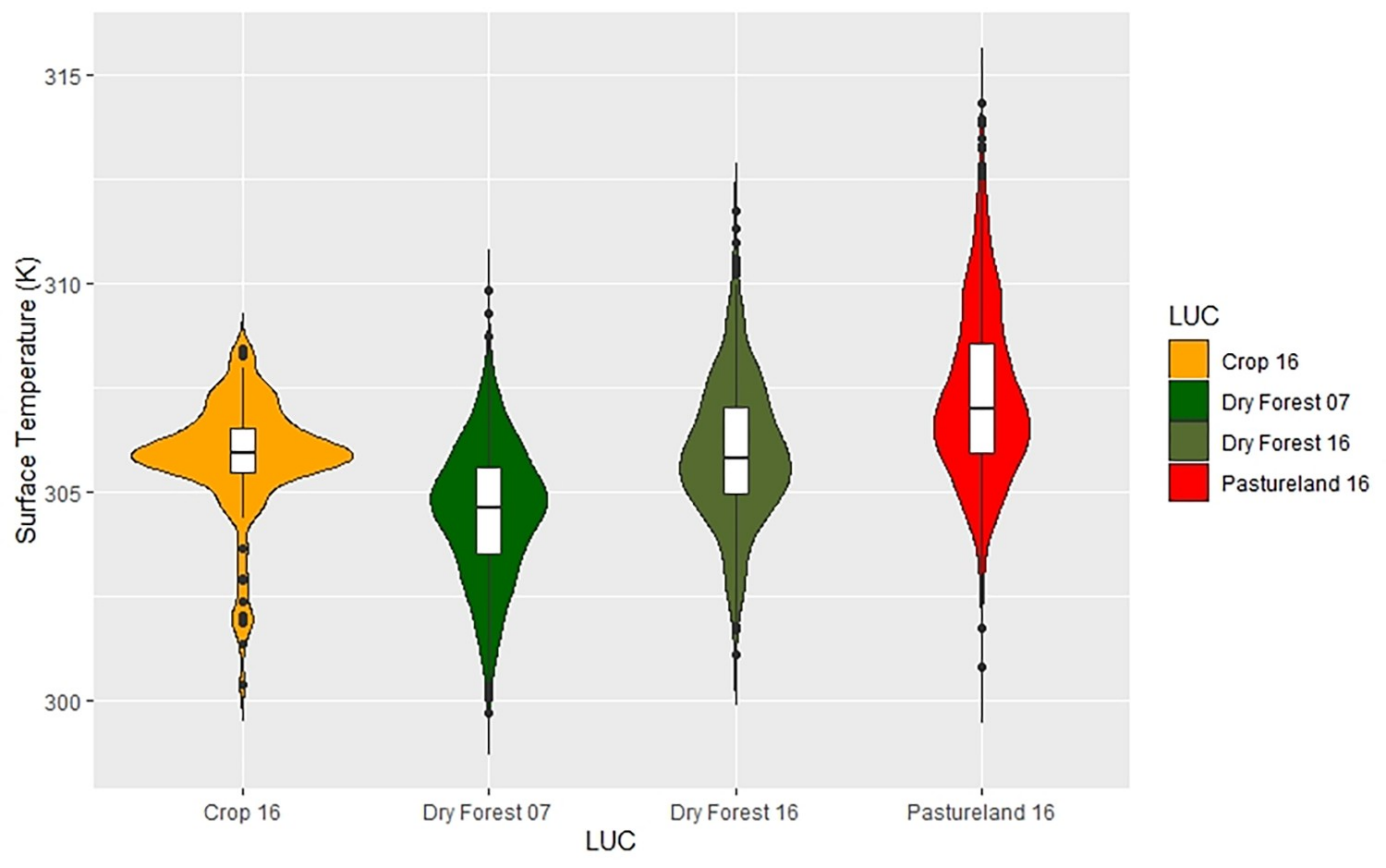
Boxplot of surface temperature (ST) considering the annual average (from the years 2007 and 2016) for the analyzed land uses and land cover.

In annual terms, in the interval of 9 years (2007 to 2016), with the verified conversions to anthropic uses, there was a substantial increase in ST (Fig 4). When converting forests into pastureland areas, the ST increased by 2.21 K ± 1.39 K, while in the conversion to crop there was an increase of 0.57 K ± 1.24 K. In this scenario, the remaining forests were warmer, with an increase in ST in the order of 1.41 K ± 1.02 K.

**Fig 5:**
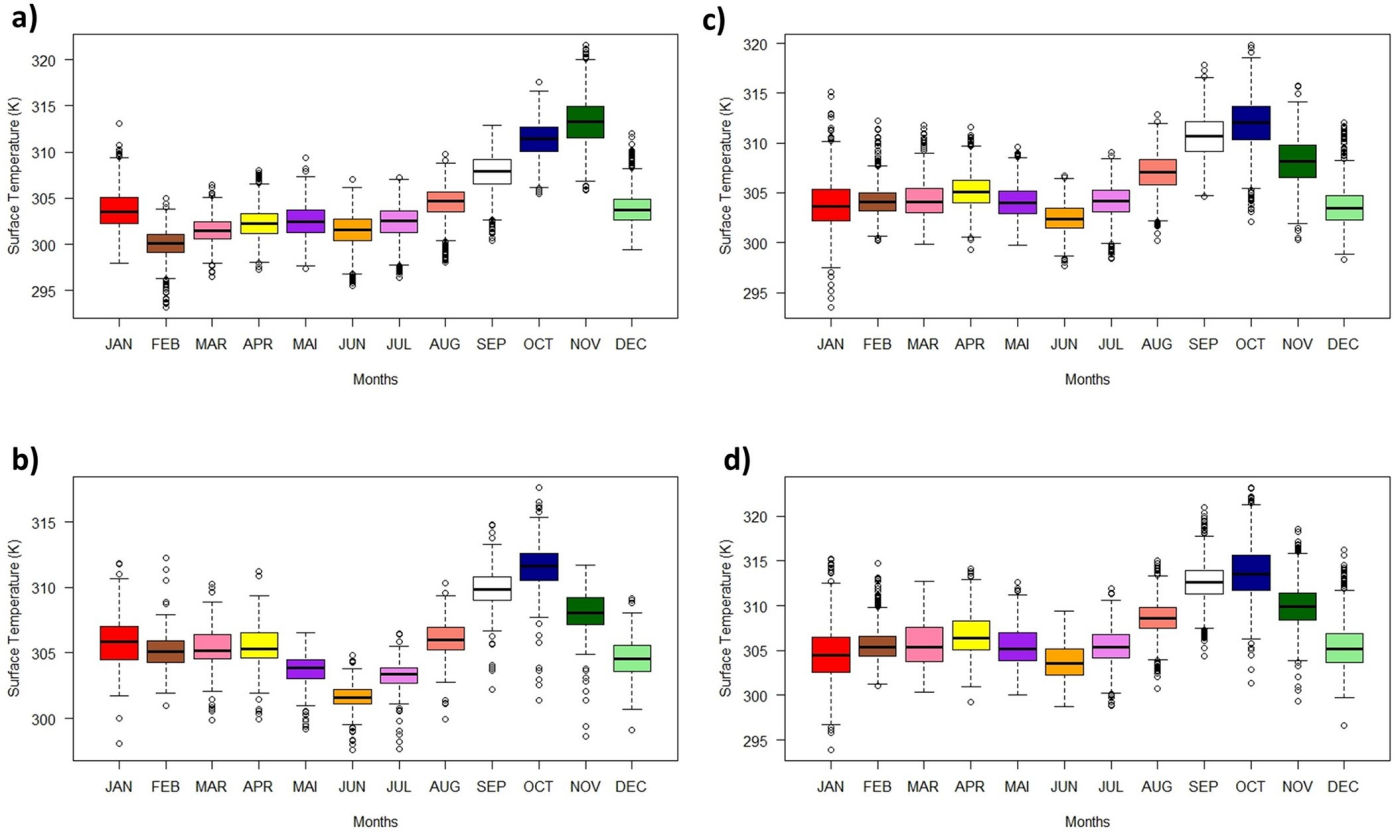
Monthly surface temperature boxplot (January to December) for land uses and land cover in the study area. a) monthly ST for the TDFs in 2007; b) monthly ST for the TDFs in 2016; c) monthly ST for pastureland in 2016; d) Monthly ST for crop in 2016.

Considering the temporal (monthly) variation of the ST for all land uses and land cover, a pattern of behavior was observed, where the highest values occurred in the months of September, October, and November (Fig 5). While the lowest values were for the months of May, June, and July. Among the analyzed domains, in most months, pastureland presented the highest ST values, with a peak in October (average of 313.8 K ± 10.4 K, reaching a maximum of 323.2 K). The TDFs areas had the highest monthly ST values, with a peak in November (313.3 K ± 8.47 K, with a maximum of 321.6 K). In 2016, this class had a ST peak in October (311.9 K ± 10.21 K). The crop class had an ST peak in October, with an average of 311.14 K ± 28.09 K and a maximum of 317.6 K. Based on the standard deviations of the classes for the months with the highest values of ST, it was evident that in 2007, in the forests domain, ST varied less (more homogeneous) in relation to the others (< standard deviation), while crop showed greater variability.

## Discussion

In this work, the behavior of ST in the domains of TDFs in the face of land use and land cover conversions in the north of the state of Minas Gerais was evaluated using remote sensing techniques. The main results found were: i) the natural domain of forest decreased by 22% between 2007 and 2016, mainly due to anthropic uses (pastureland and crop); ii) land use and land cover conversion led to an increase of 1.55 K ± 1.15 K in the 9-year interval; iii) the inclusion of pastureland at the expense of forest boosted substantial increases in ST (2.21 K ± 1.39 K).

Changes in the land use and land cover context for the domain of TDFs have been reported by several authors [23, 24, 36]. Dupin et al. [23] indicated that between 2000 and 2015 there was a net loss in the order of 3302 km^2^. The authors also concluded that cattle density (located in pastureland areas) had a direct impact on forest loss in the studied period. Regarding crops, Dupin et al. [23] also highlight that the Jaíba irrigation project (irrigated agriculture, mainly for bananas) has a high impact on the loss of TDFs in the study area. Rocha et al. [24], in monitoring land use and land cover in TDFs domains covering the north of the state of Minas Gerais to the south of Piauí (northeast region of Brazil), showed that there was forest loss in the order of 62975 km^2^ (between 2007 and 2016), in which > 72% was explained by pastureland areas, and > 20% by crop, showing similar behavior to this research, where pastureland predominated in the context of forest conversion, followed by crop.

The TDFs domains are characterized by high levels of biomass and well-structured canopy [37], substantial characteristics for the dynamics of thermal fluctuations in a given ecosystem. These factors condition the interception and absorption of solar radiation incident to the surface, as well as the conversion into water vapor fluxes, which tends to maintain low ST values (as seen in the results, Figs 4 and 5), due to surface wetting [38]. However, spatio-temporal changes in the configuration of these domains tend to quickly infer the thermal properties, especially when there is an insertion of land use with different characteristics to the natural domain of TDFs, such areas as pastures and crops, which generally have low levels of leaf area, facilitating the penetration and direct contact of solar radiation with the soil surface, consequently heating it and raising the ST, as has been reported in other studies that aimed to understand the impacts of forest systems conversions to pastureland and crop [39, 40].

Santos et al. [41], in the northwest of Brazil (state of Rondônia), showed by remote sensing techniques a difference of ST of 2.5 K in Forest areas when compared to pastureland. Pavão et al. [42], in areas in northern Brazil (Amazonas state), found an increase of 2.4 K in the conversion of forests to pastureland. Similarly, for areas of the Amazon rainforest, Querino et al. [43] observed an increase in surface temperature in the order of 6 K due to the conversion of forests to pastureland. Zhou and Wang [39], in analysis in southwest China, they found an increase of 1.5 K due to the conversion of forests into crop. In a global context, Pongratz et al. [44] observed that from the conversion of forests to pastureland and crop there was an increase in the order of 1.2 and 1.7 K respectively. While for northern Brazil, Silvério et al. [40] mention that the transition from forests to crop leads to an increase in ST in the order of 0.3 K. In addition to the increase in ST observed in view of the conversion of forests into anthropic uses between 2007 and 2016, an increase in the thermal average of the remaining forests was observed in 2016 (Fig 4). Therefore, it is believed that this may be a function of the horizontal transfer of sensible heat fluxes from the land use (pastureland/crop) (which showed higher values of ST) through advection towards the remaining TDFs in 2016. Once forests register lower values of ST, a reestablishment of the thermal balance in the face of the insertion of land use with high ΔST takes place.

In terms of implications, it is understood that the increase in ST due to the conversion of forests into anthropic uses can influence several environmental segments, especially regarding the provision of ecosystem services. ST is a proxy variable for several ecosystem services in forest areas. As forests play an intrinsic role in climate dynamics, mainly in terms of exchanges of energy flows with the atmosphere, such as latent heat and evapotranspiration [45, 46], increases in ST reflect substantial decreases in moisture levels in the surface, as it has an inversely proportional relationship with latent heat and evapotranspiration [47]. Thus, several studies that showed an increase in ST due to the conversion of forests into pastureland and crop, pointed to changes in the standards of latent heat and evapotranspiration. For example, Silvério et al. [40] recorded decreases in latent heat and evapotranspiration in the order of 32% and 35 km^3^ (in areas of Brazil), while Lathuilliere et al. [46] observed that there was a decrease in a rate of 16.2 km^3^ for evapotranspiration in areas on the western edge of Brazil. For the same region, Pongratz et al. [44] reported a 21% decrease in latent heat, while Sampaio et al. [45], in forest areas in northern Brazil, found that pastureland areas tend to reduce evapotranspiration by ~26%.

Still, in the context of ecosystem service provision, forests have a high potential for carbon (CO_2_) sequestration through net primary productivity [48, 49]. On a global scale, tropical forests store around 40% of terrestrial carbon [50], proving to be a crucial component in mitigating the effects of climate change. Specifically for TDFs, which cover ~42% of tropical forests [51], studies have found that they have high carbon stocks due to assimilation by photosynthetic processes [25]. However, as photosynthetic processes are closely linked to evapotranspiration (especially net primary productivity [52], changes in evapotranspiration can compromise the ability to assimilate atmospheric CO_2_. The exchange of flows between the forests and the atmosphere occurs in an ascending and descending manner so that CO_2_ fixation is carried out, it is necessary for the diffusion of water vapor through evapotranspiration to occur [52]. Evapotranspiration and net primary productivity have proportional behavior, therefore, as ST increases in TDFs, water vapor fluxes can decline, subsequently tending to decrease the carbon fixation capacity.

In general, we found that human actions had impacts in the context of changes (increases) in surface temperature in the domains of TDFs in the north of the state of Minas Gerais. However, although TDFs are protected by specific legislation, there is a need for extensive monitoring of forests, especially considering climatic aspects, as they are crucial for the provision of ecosystem services at different scales of approach.

## Conclusions

We found that the conversion of dry forests to pastureland and crop increased the temperature (1.55 K) in the study area (within 9 years). There was a loss of ~4,000 km^2^ (22%) of dry forests, most of which (93%) were explained by pastureland. Pastureland were also responsible for the highest values of surface temperature in 2016 (2.21 K).

Surface temperature is a crucial variable in environmental dynamics in the tropical dry forest. Therefore, considering the importance of tropical dry forests for ecosystem segments, we emphasize the need for efficient monitoring of changes in these environments, mainly by including scientific studies on climate variables in the action plans.
